# Sidewalk Conditions in Northern New Jersey: Using Google Street View Imagery and Ordinary Kriging to Assess Infrastructure for Walking

**DOI:** 10.5888/pcd16.180480

**Published:** 2019-04-16

**Authors:** Jesse J. Plascak, Adana A. M. Llanos, Laxmi B. Chavali, Cathleen Y. Xing, Nimit N. Shah, Antoinette M. Stroup, Jessica Plaha, Emily M. McCue, Andrew G. Rundle, Stephen J. Mooney

**Affiliations:** 1 Department of Biostatistics and Epidemiology, School of Public Health, Rutgers, The State University of New Jersey, Piscataway, New Jersey; 2 Rutgers Cancer Institute of New Jersey, New Brunswick, New Jersey; 3 New Jersey State Cancer Registry, Trenton, New Jersey; 4 School of Arts and Sciences, Rutgers, The State University of New Jersey, New Brunswick, New Jersey; 5 School of Environmental and Biological Sciences, Rutgers, The State University of New Jersey, New Brunswick, New Jersey; 6 Department of Epidemiology, Mailman School of Public Health, Columbia University, New York, New York; 7 Department of Epidemiology, University of Washington, Seattle, Washington

**Figure:**
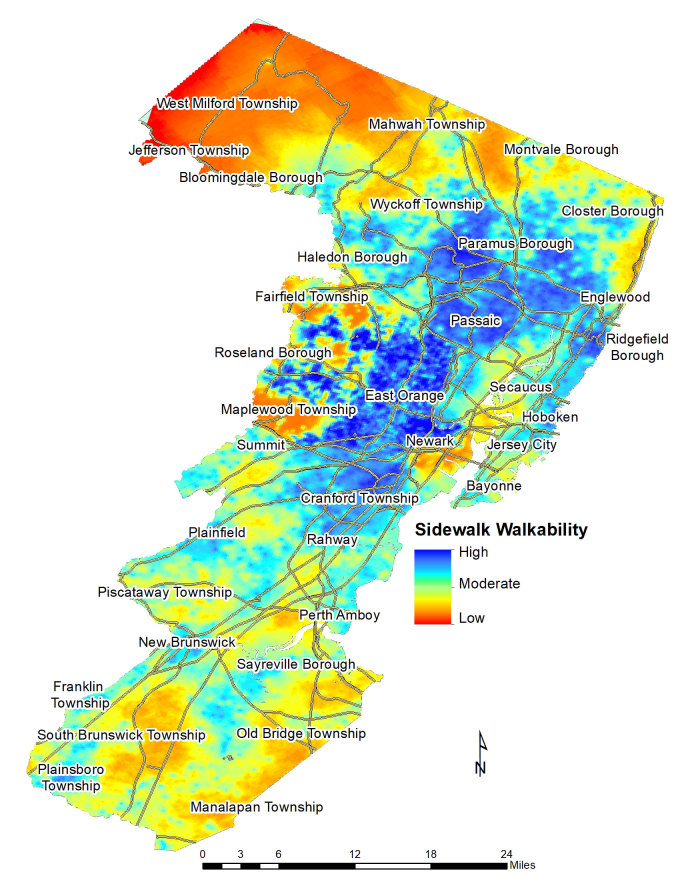
Estimated presence or absence of sidewalks and conditions of sidewalks in northeastern New Jersey. The map depicts an index of sidewalk walkability estimated from virtual street audits at 11,282 locations using Google Street View and spatial interpolation techniques. Levels of walkability ranged from low (no sidewalk or poor condition) to moderate (fair condition) to high (good condition). Precise measures of sidewalk conditions can help identify barriers to walking-based physical activity and key areas for intervention to maintain and modify sidewalk conditions.

## Background

Morbidity and mortality from chronic conditions such as obesity, diabetes, heart disease, and depression among the US population are a critical public health issue (1,2). Substantial evidence indicates that aerobic physical activity, including walking, can reduce the risk of numerous physical and mental health conditions (3,4). Walking is an excellent way to achieve the recommended amount of aerobic physical activity (≥150 minutes per week) (5). According to the Behavioral Risk Factor Surveillance System, 48.7% of US adults in 2015 did not attain the recommended amount of weekly aerobic physical activity (6).

Various built environment characteristics, including sidewalk characteristics (eg, connectivity, continuity, width, barriers, condition), could influence walkability and physical activity (7). Although street audits that observe built environment characteristics in communities are common, few studies have assessed differences in observed characteristics at the sidewalk level or address level across large, generalizable geographic areas (8). The objective of this study was to describe, in map format, sidewalk characteristics at the address level in densely populated urban and suburban areas of northeastern New Jersey, where 51.7% of adults do not participate in at least 150 minutes of weekly aerobic physical activity (6).

## Methods

We characterized sidewalks during virtual street audits via the Google Street View application, Computer Assisted Neighborhood Visual Assessment System (CANVAS) (9). Virtual street audits have been validated and demonstrated to be more cost-effective than in-person audits because of lower travel time and costs (9). We used CANVAS to assess several sidewalk characteristics, including 2 items within the 360° view at each audited location: sidewalk presence (yes or no) and sidewalk condition (poor [numerous breaks, uneven sidewalk], fair [some unevenness], or good [even, no breaks]).

We selected audited locations from non-highway roads in 6 counties in New Jersey. We selected locations approximately 150 m apart in densely populated Essex County (which encompasses Newark) and locations elsewhere approximately 600 m apart. The higher-density sampling allowed for investigation of the spatial autocorrelation structure of sidewalk characteristics and motivated the less dense sampling scheme of the 5 counties other than Essex. CANVAS auditors completed a 4-hour training session to collect data consistently for the presence or absence of sidewalks and the condition of sidewalks. Auditors were trained to report the worst sidewalk condition if sidewalks of different conditions were present at the same location. Of the 8,100 audited sidewalks observed in Essex County, 405 (5%) were rerated by each of all the auditors to provide estimates of test–retest and inter-rater agreement reliability (9). Auditors performed ratings on computers that had 2 monitors: one monitor displayed data input forms and the second monitor displayed the Google Street View scene. We downloaded and analyzed data on completed ratings; 11,282 locations were available for analysis.

Data on sidewalk presence and condition were combined into a sidewalk walkability variable with the following possible ordinal values: 0 (no sidewalk), 1 (poor sidewalk condition), 2 (fair sidewalk condition), and 3 (good sidewalk condition). Test–retest and inter-rater reliability were high in the reliability subsample (all intraclass correlation coefficients ≥0.89). Spatial analyses indicated that measured sidewalk walkability values correlated with other values at locations separated up to 4,200 m (2.6 miles). We used ordinary kriging to estimate sidewalk walkability values across the study area (9). Kriging models are spatial interpolation methods that predict sidewalk walkability at nonaudited locations based, in part, on the observed similarity between walkability values assessed at audited locations. Ordinary kriging results in continuous predictions, and we plotted these continuous predictions on a map as a range of walkability, from low (no sidewalk or poor condition) to moderate (fair condition) to high (good sidewalk condition). We analyzed concurrent validity of the sidewalk walkability construct through a census tract–level Spearman correlation coefficient (ρ = 0.22, *P* < .001) of the relationship between average sidewalk walkability in each tract and proportion of commuters in that tract who reported walking to work in the 2012–2016 American Community Survey (10). We used SAS version 9.4 (SAS Institute Inc) and ArcGIS version 10.5 (Esri) in all analyses.

## Main Findings

We found several geographic patterns in sidewalk walkability in northern New Jersey. The presence of any sidewalk and the presence of sidewalks in fair or good condition were more common in urban cores (Newark, East Orange, Passaic, and Hoboken) than outside these cores and occurred less frequently as distance from these cores increased (for example, in northern West Milford Township, northern Mahwah Township, and southern Manalapan Township). However, we found heterogeneity in sidewalk walkability at a smaller geographic scale, which was subtle in the urban cores but more apparent in the western suburbs of Newark and East Orange (for example, in Roseland Borough). Generally, sidewalks were absent or in poor condition along major roads in otherwise walkable urban cores.

## Action

We used virtual street audits and spatial interpolation techniques to construct a detailed high-resolution map of sidewalk conditions in northeastern New Jersey. Such high-resolution maps can be informative and powerful tools, offering finer-grain detail on sidewalk conditions than would be available in tabular format or a choropleth map. We demonstrated that the use of innovative, spatially based sampling and estimation methods, publicly available Google Street View scenery, and the CANVAS application can allow for large-scale, routine, and standardized collection of variables related to sidewalk characteristics. Such information can be useful both for research and practice. For researchers, precise measures of sidewalk conditions can help identify barriers to walking-based physical activity. For practitioners, this map may help identify key areas for intervention to maintain and modify sidewalk conditions (11). Improvements made to walkability may be one of the most cost-effective strategies for increasing physical activity and reducing disparities in chronic disease, particularly among populations that do not achieve recommended amounts of physical activity (4). A map indicating regions for improvement in walkability may facilitate identification of regions in need of sidewalk improvements to support walking-based physical activity. Future research should extend this measure across all of New Jersey and further explore potential correlates of sidewalk conditions, such as race/ethnicity and socioeconomic status.

## References

[1] Ward BW , Schiller JS , Goodman RA . Multiple chronic conditions among US adults: a 2012 update. Prev Chronic Dis 2014;11:E62. 10.5888/pcd11.130389 24742395 PMC3992293

[2] Pratt LA , Brody DJ . Depression and obesity in the U.S. adult household population, 2005–2010. NCHS Data Brief 2014;(167):1–8. 25321386

[3] US Department of Health and Human Services. 2018 Physical Activity Guidelines Advisory Committee scientific report. Washington (DC): US Department of Health and Human Services; 2018.

[4] US Department of Health and Human Services. Step it up! The Surgeon General’s call to action to promote walking and walkable communities. Washington (DC): Office of the Surgeon General; 2015. 30860691

[5] Garber CE , Blissmer B , Deschenes MR , Franklin BA , Lamonte MJ , Lee IM , American College of Sports Medicine position stand. Quantity and quality of exercise for developing and maintaining cardiorespiratory, musculoskeletal, and neuromotor fitness in apparently healthy adults: guidance for prescribing exercise. Med Sci Sports Exerc 2011;43(7):1334–59. 10.1249/MSS.0b013e318213fefb 21694556

[6] Centers for Disease Control and Prevention. Behavioral Risk Factor Surveillance System prevalence and trends data: 2015. https://www.cdc.gov/brfss/brfssprevalence. Accessed August 7, 2018.

[7] Cerin E , Saelens BE , Sallis JF , Frank LD . Neighborhood Environment Walkability Scale: validity and development of a short form. Med Sci Sports Exerc 2006;38(9):1682–91. 10.1249/01.mss.0000227639.83607.4d 16960531

[8] Lopez RP , Hynes HP . Obesity, physical activity, and the urban environment: public health research needs. Environ Health 2006;5(1):25. 10.1186/1476-069X-5-25 16981988 PMC1586006

[9] Mooney SJ , Bader MDM , Lovasi GS , Teitler JO , Koenen KC , Aiello AE , Street audits to measure neighborhood disorder: virtual or in-person? Am J Epidemiol 2017;186(3):265–73. 10.1093/aje/kwx004 28899028 PMC5860155

[10] US Census Bureau. American Community Survey, 2012–2016. American Community Survey 5-year estimates; using American FactFinder. 2018. http://factfinder2.census.gov. Accessed August 30, 2018.

[11] Zandieh R , Flacke J , Martinez J , Jones P , van Maarseveen M . Do inequalities in neighborhood walkability drive disparities in older adults’ outdoor walking? Int J Environ Res Public Health 2017;14(7):E740. 10.3390/ijerph14070740 28686219 PMC5551178

